# How maternal position affects umbilical and middle cerebral artery Doppler indices: insights from a scoping review

**DOI:** 10.61622/rbgo/2025rbgo45

**Published:** 2025-07-15

**Authors:** Larissa Raso Hammes, Andre Hadyme Miyague, Renato Mitsunori Nisihara

**Affiliations:** 1 Universidade Federal do Paraná Curitiba PR Brasil Universidade Federal do Paraná, Curitiba, PR, Brasil.; 2 Clínica Vous – Medicina Fetal e Reprodutiva Curitiba PR Brasil Clínica Vous – Medicina Fetal e Reprodutiva, Curitiba, PR, Brasil.

**Keywords:** Pregnancy, Vena cava, inferior, Middle cerebral artery, Supine position, Patient positioning, Fetal growth retardation, Hypoxia, Ultrasonography, doppler, Fetal ultrasound, Fetal doppler, Fetal brain-sparing effect

## Abstract

**Background:**

During pregnancy, the maternal supine position may reduce uterine and placental perfusion due to compression of the aorta and inferior vena cava by the gravid uterus, potentially impairing maternal and fetal oxygenation.

**Objective:**

This scoping review aimed to summarize the available evidence on the impact of maternal positioning during ultrasound examinations on fetal Doppler indices of the umbilical artery (UA) and middle cerebral artery (MCA).

**Methods:**

Studies were eligible if they included pregnant women undergoing fetal Doppler assessment in at least two different maternal positions and reported outcomes related to UA and/or MCA indices. Only studies published in English in the past 20 years were included.

**Sources of evidence:**

A comprehensive literature search was conducted in the PubMed/MEDLINE, Web of Science, and Scopus databases between September and October 2023.

**Charting methods:**

Two independent reviewers conducted the initial screening for relevance, with conflicts resolved by consensus or by a third reviewer.

**Results:**

Thirteen studies were initially identified. After applying the eligibility criteria, six observational prospective studies were included. These studies assessed changes in UA-PI, MCA-PI, and/or the cerebroplacental ratio (CPR) in response to different maternal positions during Doppler ultrasound.

**Conclusions:**

Evidence suggests that maternal positioning during fetal Doppler ultrasound can influence arterial indices, particularly when comparing supine and lateral decubitus positions. However, variability in methodology and small sample sizes limit the generalizability of findings. Further standardized studies are needed to guide clinical recommendations.

## Introduction

For the past decades, Doppler ultrasound has been an essential tool to access feto-placental circulation and therefore estimate fetal wellbeing allowing better clinical management especially in cases of fetal growth restriction (FGR). Such importance is clear as Doppler indices (e.g. pulsatility and resistance indices) obtained by the velocity quantification of fetal-placental circulation have been closely related to perinatal outcome.^(1,2)^

The compression of maternal inferior vena cava (IVC) against the spine determined by maternal supine position (MSP), mainly in the third trimester, is a well-known effect associated with maternal blood hypotension and hence decreased blood flow to the uterus, placenta and fetus.^(3-8)^ Like so, for over 70 years it has been standard of care practice to gently tilt the pregnant to left side by placing a wedge at the back of the patient during labor or surgical procedures in order to displace the uterus from the IVC and thus improve maternal hemodynamics and oxygenation. The maternal aorta-cava compression effect caused by MSP upon fetal hemodynamics and vitality has already been extensively described elsewhere^(9,10)^ as fetal adaptation to mild hypoxic stress caused by MSP includes increased blood flow through the fetal middle cerebral artery, increased fetal inactivity, and a greater number of nonreactive fetal heart rate traces.^(11-14)^

However, the presumption that MSP itself influences uterine perfusion and consequently altering Doppler signaling quantification may be subject to many sources of bias inherent to the technique itself such as the increasing gestational age, maternal body mass index (BMI), maternal preexisting conditions, fetal vascular adaptation to MSP throughout time, the degree of vena caval compression etc.^(7,15)^

Nevertheless, despite the logical claim that MSP influences Doppler signaling quantification, we were not able to find any mention/recommendation of maternal position during Doppler ultrasound examination in any major paper/consensus/protocols related in the past ten years.^(16-23)^ Additionally, we were not able to find in any online available related medical text any standardized protocol to acquire Doppler velocimetry in cases where MSP could alter the result.

This review addresses the existing gap in the literature by providing a thorough and insightful review of the influence of maternal position during ultrasound exam on umbilical and middle cerebral artery fetal Doppler indices and its implications for clinical practice.

## Methods

To guide the study search and selection, the following research question was formulated: "How does maternal positioning during ultrasound examinations impact fetal Doppler parameters?" This question was developed using the Population, Concept, and Context (PCC) strategy.^(24)^ Therefore, "P" refers to pregnant women at term. "C" denotes the concept of maternal positioning during ultrasound Doppler exams. The final "C" encompasses the context of clinical settings, aiming to investigate the potential changes in Doppler parameters based on different maternal positions to inform prenatal care practices.

This scoping review protocol was registered in the Open Science Framework (OSF) (registration DOI: https://doi.org/10.17605/OSF.IO/C3PVM).

A comprehensive literature search was conducted in the PubMed/MEDLINE, Web of Science, and Scopus databases between September and October 2023. Studies were retrieved using a combination of Medical Subject Headings (MeSH) and free-text terms related to maternal positioning and fetal Doppler assessment. The following search string was used: ("supine position*"[Title/Abstract] OR "lateral decubitus"[Title/Abstract] OR "maternal position"[Title/Abstract]) AND ("Doppler indices"[Title/Abstract] OR "cerebroplacental ratio"[Title/Abstract] OR "brain sparing"[Title/Abstract] OR "fetal circulation"[Title/Abstract] OR "cardiac index"[Title/Abstract]). Searches were limited to articles written in English and published in the last 20 years. Only studies involving pregnant women undergoing Doppler ultrasound in at least two different maternal positions were considered eligible.

The titles and abstracts retrieved from the databases were imported into the Rayyan systematic review platform to facilitate the screening process. Two independent reviewers conducted the initial screening for relevance, with conflicts resolved by consensus or by a third reviewer. Full texts of potentially eligible studies were then reviewed for inclusion. The review was conducted according to the guidelines outlined by the Joanna Briggs Institute (JBI) for scoping reviews.^(25)^ The JBI guidelines were adapted to fit the specific objectives and scope of this study. A customized data extraction form was developed based on these guidelines to systematically capture relevant information from each included study, such as study characteristics, population details, maternal positioning protocols, Doppler parameters assessed, and key findings. This study followed the guidelines and checklist of the Preferred Reporting Items for Systematic reviews and Meta-Analyses extension for Scoping Reviews (PRISMA-ScR), developed under the guidance of the EQUATOR network (Enhancing the Quality and Transparency Of health Research).^(26)^

## Results

**Figure 1:**
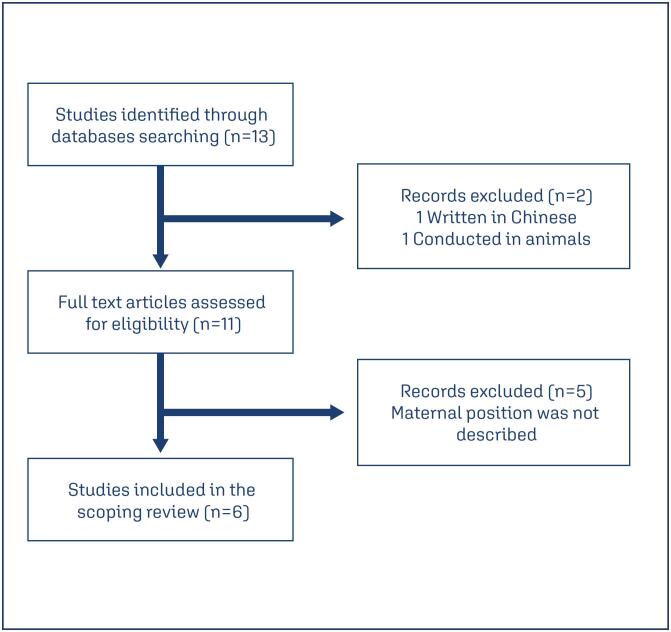
Flowchart of the selected studies

A total of 13 potential studies were initially identified. Two studies were excluded: one because was written in Chinese and the other was conducted on animals. Full-text reading of the remaining studies resulted in the exclusion of five studies that did not directly address a change in maternal position during the ultrasound exam or did not describe which position the exam was performed in. Using the outlined methodology, the systematic literature review identified six studies that fulfilled the inclusion criteria - assessment of CPR, UA-PI, and/or MCA-PI and a change in maternal position during the exam. Figure 1 shows the flowchart of the selection process, including numbers of studies screened, excluded, and reasons for exclusion.

A detailed summary of the characteristics of the included studies is provided in Chart 1, including study design, sample size, maternal positioning protocols, Doppler parameters evaluated, and main outcomes. All included studies had an observational prospective design. One study assessed the influence of maternal supine position during spinal anesthesia for elective cesarean section, while the remaining five studies assessed different Doppler parameters related to maternal positioning, in either the lateral decubitus or supine position.

**Chart 1:** Summary of the characteristics and findings of the included studies

Author	Year	Country/Sample size	Purpose	Findings	Observations
Khatib et al.^(7)^	2014	Israel n=23	To examine whether fetal blood redistribution can be elicited by a physiological stress associated with maternal position in low-risk pregnancies.	MCA-PI and UA-PI were obtained following 15 min rest in the left lateral decubitus position. Subsequently, the women were asked to change into the supine position for an additional 15 min and Doppler measurements were repeated. Five good quality flow velocity waveforms were obtained from each vessel. The UA-PI and MCA-PI decreased after changing from left lateral to supine position.	This was the only study to have shown that UA-PI declined by changing from left lateral to supine position and it is hypothesized to happen due to a compensatory mechanism aimed at improving feto-placental perfusion during a sudden drop in uteroplacental blood flow.
Robertson et al.^(27)^	2020	Australia n=274	To correlate fetal hemodynamics in a cohort of women with apparently uncomplicated pregnancies with their nocturnal sleep position.	The MCA-PI and the CPR were significantly lower in the supine position in the sleep cohort. It was not indicated how many waveforms were evaluated for each vessel	The authors’ findings provide further evidence of some potential physiological mechanisms underpinning the increased perinatal risks associated with the supine sleep position, including perinatal death, even in appropriately grown fetuses.
Silva et al.^(28)^	2016	Brazil n=20	To examine whether fetal blood circulation is influenced by the maternal position and for how long this effect persists during an ultrasound examination.	Five good quality flow velocity waveforms were evaluated for each vessel. Changing the maternal position from the left lateral to the supine position caused a reduction in MCA-PI after 5 min. This reduction lasted less than 10 min. There was no significant difference between the left lateral and the supine position at 5 and 10 min in terms of UA-PI.	A limitation of this study was the absence of an assessment for maternal or fetal oxygen saturation. That could have clarified the potential relationship between lower oxygen tension in the mother's blood due to supine position - which affects maternal lung function in late pregnancy - and reduced fetal oxygenation. This reduction is presumed to prompt a compensatory mechanism, resulting in a temporary increase in cerebral perfusion.
McLaren et al.^(29)^	2020	USA n=75	To assess whether there is a change in fetal MCA-PI associated with maternal position in both normal pregnancies and pregnancies with factors associated with placental pathologies.	Doppler indices were calculated using three uniform waveforms. The MCA-PI did not change significantly between the lateral and supine positions in high-risk pregnancies, while it decreased in low-risk pregnancies. This difference was observed even in the absence of clinical findings (e.g. growth restriction or oligohydramnios).	The findings point to an attenuated ability of fetuses in high-risk pregnancies to respond to physiologic stress since they are under constant physiological stress and are incapable of further accommodation and/or have a desensitized response to acute hypoxia caused by maternal position due to the chronic hypoxic environment.
Armstrong et al.^(30)^	2011	UK n=25	To measure cardiac index, UA IP and FHR in 4 different positions during neuraxial blockade in women with uncomplicated pregnancies present for elective cesarean delivery at term.	5 consecutive waveforms were evaluated, and no significant difference was found in fetal heart rate and in AU-PI among the 4 positions.	The authors also found that maternal position in the flexed sitting versus lateral decubitus changed minimally maternal hemodynamic variables in the healthy, term, nonlaboring maternal-fetal dyad. Further research is required to evaluate the effects of positioning in women with cardiovascular diseases or uteroplacental insufficiency.
Wu et al.^(31)^	2023	China n=150	To evaluate the effect of supine, right lateral, and left lateral positions on UA and MCA-PI in low-risk singleton full-term pregnant women.	There were no significant differences in both UA-PI and MCA-PI among the three maternal positions.	

## Discussion

The findings from this study show that inadequate uteroplacental blood flow has been associated with fetal growth restriction, having a negative impact on fetal growth and therefore altering Doppler parameters. Depending on the duration of the ultrasound exam, the position in which the patient performs the exam may become relevant. However, despite the significant implications of these findings, current ultrasound guidelines^(16,18,20,23)^ do not specify the maternal positions in which Doppler measurements should be obtained. Although the dorsal decubitus position is widely adopted in clinical practice, there is a lack of clear guidance on how this position may affect fetal Doppler results.

In 2014, Khatib et al.^(7)^ were the first to show that the occurrence of fetal centralization may be associated with physiological stress situations, such as maternal positioning, in low-risk pregnancies. The researchers assessed the effect of changing the maternal position from left lateral decubitus to supine for 15 minutes on the recording of Doppler parameters during third-trimester low-risk pregnancies. Twenty-three low-risk pregnant women between 36 to 40 week's gestation participated in the study. For each woman, Doppler flow measurements were acquired from the MCA and UA following a 15-minute period of rest in the left lateral decubitus position. Subsequently, the women were instructed to transition to the supine position for an additional 15 minutes to allow for fetal-maternal adaptation, after which Doppler measurements were repeated. To minimize variability between observers, Doppler measurements were conducted by a single operator. At least five high-quality flow velocity waveforms were captured from each vessel and three separate measurements were taken and averaged to determine the final value. Results showed a decrease in the MCA-PI and UA-PI after changing the position from left lateral decubitus to supine position. This shows that the fetal centralization phenomenon can be triggered by a physiological acute stress situation such as a sudden drop in uteroplacental perfusion due to aortocaval compression. Robertson el al. (2020)^(27)^ found similar results when examining the relationship between the position pregnant women reported to assume during sleep and doppler parameters. 274 women underwent a single ultrasound assessment from 35 weeks of gestation and completed a sleep survey, which asked women to describe their sleep position both going to sleep and waking from sleep before they were pregnant, over the last one month of pregnancy, over the last week of pregnancy, and the night prior to completing the questionnaire. Fetal Doppler measurements – MCA-PI and UA-PI – were recorded in triplicate over multiple cardiac cycles in the absence of maternal or fetal breathing movements with the average values reported. The authors reported that both doppler index were significantly lower in the supine position compared to the left lateral decubitus (LLD) – consistent with the phenomenon of fetal brain-sparing. Unlike Khatib et al.,^(7)^ Robertson et al.^(27)^ did not find differences in UA-PI regarding maternal positioning.

Silva et al.^(28)^ investigated whether fetal circulation is influenced by the maternal supine position during ultrasound examination. Initially, each of the 20 women recruited were instructed to assume the left lateral position for 5 minutes, during which measurements were taken for fetal biometry, amniotic fluid index, MCA-PI and UA-PI. Afterwards, the women were directed to switch to the supine position, and Doppler measurements for the fetus were repeated at 5 minutes and 10 minutes. For each vessel, a minimum of five high-quality flow velocity waveforms were graphed. Silva et al.^(28)^ found no significant differences in the UA flow with the change in position, and a decrease in MCA-PI after the first 5 minutes in the supine position. Their study also evaluated the length of time that the patient remained in each position during the Doppler examination and found that the reduction in MCA-PI did not persist after 10 minutes in the supine position, leading to the conclusion that a change in position is sufficient to cause a transient increase in cerebral perfusion. These findings contrast with those of Khatib et al.^(7)^ who found that the change in MCA-PI was sustained after 15 minutes in the supine position, in addition to variations in AU-PI with the change in maternal position.

McLaren et al., in 2020,^(29)^ were the first to include high-risk pregnant women with placental pathologies to evaluate MCA-PI in various maternal positions. The participants were categorized into two groups: high-risk pregnant women with conditions associated with placental pathology (i.e. pregnancies characterized by abnormal plasma protein A; β-human chorionic gonadotropin; inhibin A; estriol and/or α-fetoprotein levels, chronic hypertension and pre-gestational diabetes) (study group, n=41) and low-risk pregnant women (control group, n=34). MCA-IP was obtained in the maternal supine position and then the patient was positioned in the left lateral decubitus for at least 5 min before it was repeated. Doppler indices were calculated using three uniform waveforms. It was found that the MCA-IP value increased when switching from supine to left lateral decubitus in the low-risk group, but not in the high-risk group. The authors concluded that conditions associated with placental pathologies may lead to chronic placental hypoxia and an increased risk of adverse perinatal events. Additionally, in high-risk pregnancies with placental pathologies, the fetus was constantly under physiological stress, and the "brain-sparing" mechanism may become desensitized to hypoxia. This study also differed from others, as it assessed pregnant women in their second trimester, from 18 weeks onwards (the average gestational age were 28.8 weeks in the control group and 30.2 weeks in the study group). In addition to considering the presence of placental pathologies, gestational age can also have an influence on changes in Doppler measurements, as earlier pregnancies can cause less compression on the vena cava and cause less maternal and fetal hemodynamic changes.

Armstrong et al.^(30)^ assessed maternal cardiac output and fetal well-being in four maternal positions during the procedure of spinal anesthesia for elective caesarean section in 35 women presenting for elective caesarean delivery with singleton pregnancies at term. In each position - supine with a 15-degree tilt to the left, sitting with neck and hips flexed, flexed right lateral, and flexed left lateral – the patient was allowed to rest for 5 minutes before any measurements were taken and then 10 minutes were allocated for acquiring suprasternal Doppler readings, followed by an additional 5 minutes for obtaining fetal blood flow readings. If satisfactory signal acquisition couldn't be achieved within these time limits, the patient was excluded from analysis. Fetal doppler data for each maternal position comprised the average of 3 measurements derived from the average of 5 consecutive cardiac cycles. Results showed that the degree of aortocaval compression during anesthesia did not affect fetal well-being, since there was no significant difference in fetal heart rate and UA flow among the four proposed positions. Wu et al.^(31)^ conducted a study with low-risk pregnancies between 37 and 40 weeks to assess fetal Doppler in the left lateral and supine position. Consistent with Armstrong et al.,^(30)^ there was no statistically significant difference between the left lateral, right lateral, and supine positions in both UA-PI and MCA-PI.

Three of the four studies that analyzed UA-PI, found no significant differences in UA-PI between maternal positions^(28,30,31)^ and one found a decrease in UA-PI after switching from the left lateral to the supine position.^(7)^ In 1991, Van Katwijk and Wladimiroff^(11)^ found that although all PI values from the umbilical artery were situated within the normal range with changes maternal positions during the ultrasound exam - standing (position I), supine (position II), standing (position III), and supine (position IV), a significantly higher PI was observed in the supine position when compared to the standing position, suggesting an increased umbilical placental vascular resistance with the mother lying on supine position. These findings raise the question of whether additional factors, such as maternal weight, neural factors, maternal comorbidities, and pre-existing placental insufficiency, impact the flow in the umbilical artery in addition to the mother´s position. The supine decubitus position can cause subtle changes in fetal circulation, independent of any association with growth disorders, which could increase the fetuses’ vulnerability to adverse perinatal events.

Despite the well-established concept that the compression of the inferior vena cava and the abdominal aorta reduces uterine and placental blood flow,^(8,32)^ potentially leading to fetal "brain-sparing" phenomenon,^(33-37)^ the hypothesis of whether the supine maternal position could induce pathological changes in fetal hemodynamics similar to those observed in FGR secondary to placental insufficiency has been raised. Previous research has established that the supine sleeping position is a modifiable risk factor for late stillbirth.^(38-40)^ Anderson et al.^(14)^ have shown that sleeping in a supine position for 1 to 4 weeks during late pregnancy was associated with a significant decrease in birth weight, with a 10-percentile drop in birth weight centiles. Individuals who regularly slept in this position also had a threefold higher risk of small for gestational age (SGA) fetuses, regardless of any other causes. Notably, other sleeping positions, such as left and right decubitus or other sleeping positions showed similar birth weights and centiles. This suggests that the supine position's effect on maternal cardiac output and fetal blood supply contributes to a significant clinically independent reduction in birth weight.

Although evidence suggests that maternal position can influence fetal Doppler indices, inconsistencies in the literature may arise from methodological limitations, such as the absence of gestational age-specific centiles and variability in the published reference ranges for umbilical artery Doppler measurements.^(41,42)^ These inaccuracies can lead to misclassification of Doppler indices, compromising the reliability of study findings and potentially resulting in inappropriate clinical interventions.^(43)^ Additional studies are needed to evaluate the influence of maternal positioning using standardized reference charts for UA and MCA-IP and considering factors such as the duration of each position during the exam, examiner expertise, gestational age, and maternal comorbidities that may contribute to placental pathologies.

Understanding the potential alterations in Doppler indices due to maternal positioning allows clinicians to make more informed decisions, potentially prevent misdiagnoses that could determine a growth-restricted or misdiagnosed fetuses with diminished adaptive mechanisms due to placental pathologies, and therefore placing them at risk of adverse perinatal outcomes.

## Conclusion

Despite the well-known fact that the gravid uterus compresses the aorta and inferior vena cava in the supine position, which can lead to changes in fetal circulation, our research reveals conflicting results in the literature regarding the impact of maternal position on umbilical and middle cerebral artery fetal Doppler indices. Some studies indicate significant changes, while others do not, highlighting the need for further investigation. Importantly, no current guideline provides clear recommendations on the optimal maternal position during the ultrasound exam, and yet there is no sufficient evidence to determine whether maternal position significantly affects Doppler results to the extent that it would alter fetal diagnosis, such as reclassifying a fetus from small for gestational age to FGR. This review underscores the necessity of conducting additional studies and revising existing guidelines on the influence of maternal position on umbilical and middle cerebral artery Doppler indices for accurately diagnosing and managing FGR, as current parameters may be biased.
